# Occupational Risks in Hospitals, Quality of Life, and Quality of Work Life: A Systematic Review

**DOI:** 10.3390/ijerph182111434

**Published:** 2021-10-30

**Authors:** Carlos Rodrigo Nascimento de Lira, Rita de Cássia Akutsu, Priscila Ribas de Farias Costa, Luana de Oliveira Leite, Karine Brito Beck da Silva, Raquel B. A. Botelho, António Raposo, Heesup Han, Antonio Ariza-Montes, Luis Araya-Castillo, Renata Puppin Zandonadi

**Affiliations:** 1 School of Nutrition, Federal University of Bahia, Avenida Araújo Pinho, n°32, Canela, Salvador CEP: 40110-150, Brazil; carlos.rodrigo.n@hotmail.com (C.R.N.d.L.); priscilarf@yahoo.com.br (P.R.d.F.C.); luanaleite_nutri@yahoo.com.br (L.d.O.L.); nutkarinebeck@hotmail.com (K.B.B.d.S.); 2 Department of Nutrition, Campus Darcy Ribeiro, University of Brasilia, Asa Norte, Distrito Federal, Brasília CEP: 70910-900, Brazil; rita.akutsu@gmail.com (R.d.C.A.); raquelbotelho@unb.br (R.B.A.B.); renatapz@unb.br (R.P.Z.); 3 Department of Life Sciences, Bahia State University, Rua Silveira Martins, Cabula, Salvador CEP: 41150-000, Brazil; 4 CBIOS (Research Center for Biosciences and Health Technologies), Universidade Lusófona de Humanidades e Tecnologias, Campo Grande 376, 1749-024 Lisboa, Portugal; 5 College of Hospitality and Tourism Management, Sejong University, Seoul 05006, Korea; 6 Social Matters Research Group, Universidad Loyola Andalucía, C/Escritor Castilla Aguayo, 414004 Córdoba, Spain; ariza@uloyola.es; 7 Faculty of Business Administration, Universidad Autónoma de Chile, Santiago 7500912, Chile; 8 Facultad de Economía y Negocios, Universidad Andrés Bello, Santiago de Chile 7591538, Chile; luis.araya@unab.cl

**Keywords:** systematic review, quality of work life, occupational risks

## Abstract

This study systematically reviewed the relationship between occupational risks and quality of life (QoL) and quality of work life (QWL) in hospitals. A systematic review was performed according to the recommendations of the Preferred Reporting Items for Systematic Reviews and Meta-Analyses guide, and the protocol was submitted on the PROSPERO website (CRD 2019127865). The last search was performed in June 2021 by two independent reviewers in the main databases, a gray literature database, and a manual search (LILACS, MEDLINE/PubMed, PsycINFO, CINAHL, Scopus, Embase, Brazilian Digital Library of Theses and Dissertations, Ovid). As eligibility criteria, we included observational studies, with adult hospital workers, with no restrictions on date and language, any type of instrument to assess QoL and QWL, any definition of QoL and QWL, and studies that presented the relationship between exposure and outcome. Newcastle–Ottawa was used to assess the methodological quality and RTI-Item Bank to assess the risk of bias. Given the impossibility of performing a meta-analysis, a qualitative synthesis was used to present the results. Thus, 11 studies met the criteria and were included in the review, with 6923 individuals aged 18 to 64 years. The studies were mainly carried out with health professionals (81.81%), women (63.60%), and in Asian countries (63.63%). All studies used different instruments and ways to categorize the QoL and QWL, and occupational risks. Only one study assessed occupational noise and another the ergonomic risk. All of them presented a relationship between occupational risk and quality of work life. They pointed to the need for measures to improve the lives of these professionals in the work environment. Therefore, studies show a relationship between occupational risks (noise, ergonomics, and stress) and workers’ perception of low or moderate quality of work life. However, more homogeneous studies are necessary for instruments, conceptualization, and categorization of quality of work life.

## 1. Introduction

In hospital work, professionals face the dilemma of specialized activity, with inadequate labor conditions and a favorable environment for occupational risks, among other factors. These characteristics, associated with the precarious labor context observed in the world, mainly because of the outsourcing process, flexibilization, and meritocracy, are responsible for workers always believing that they are liable for everything that happens in their work [1,2,3,4,5].

It is further observed that this environment is a space of remarkable human interaction. Such interaction occurs in a complex way due to hierarchical relationships between the professionals’ groups involved [6,7]. In addition, hospital work is marked by rules, routines, and compliance with protocols due to the type of service, which Santos [6] considered an organization-prescribed activity.

Given the dynamics of work in hospitals, this is a highly favorable environment for occupational hazards. In this study, occupational risks are understood as work situations that can disrupt workers’ physical, mental, and social balance, such as occupational stress, physical, chemical, biological, ergonomic, and accident risks [8,9,10]. For Silva [11], these occupational risks are responsible for causing injury to the production, quality, care provided, and workers’ health.

The multiplicity of these risks in hospitals will occur to a greater or lesser extent, determined by the sector within the organization. Thus, these risks are present in various activities and can cause severe and numerous problems for workers [12]. The concern with the health of these professionals started to gain notoriety as evidence emerged that they are more susceptible to injuries with occupational hazards than other professionals [10,13]. Given the direct relationship between occupational risks and health conditions, there is a strong influence on both the quality of life (QL) and the quality of work life (QWL) of these professionals [14].

In 1995, the World Health Organization [15] defined quality of life as “the individual’s perception of their insertion in life in the context of the culture and value systems in which they live and with their goals, expectations, standards, and concerns”. Therefore, there is multidimensionality in the term, and it permeates numerous areas of the individual’s life, including work, a place that sometimes seems to be intrinsic to human life. Thus, QWL derives from this broader construct and denotes a way of recognizing the relationships between life and the demands of the work environment, or even how organizational issues affect workers’ physical and/or mental health [16,17,18].

The fact that work is central in the lives of individuals and that many years of life are lived within organizations, the dimension of work in quality of life has great importance, and it is not easy to dissociate them [19].

Several theoretical models related to the quality of life at work are identified. The model founded by Nadler and Lawler [20] proposes ways of thinking about the worker and the company to have satisfactory results. Based on this theoretical model, the work environment must be favorable for health protection, insurance, and favoring the well-being of workers, and for the achievement of such measures, occupational risks should be considered to guarantee physical and mental integrity [21,22]. Thus, QWL is a concept that is related to working conditions, and favorable working conditions are recognized as the most cited QoL indicators [23].

Studying these risks and the relationship with QOL and QWL becomes necessary to understand how they are configured and related, as providing a safe work environment allows the establishment of efficient protection and safety measures. Furthermore, analyzing the articulations between occupational risks, QOL, and QWL helps to understand workers’ perception about their work environment and, at the same time, contributes to the development of science, theory, and practice. Such systematic investigation also clarifies the gaps and shows the need for studies on this scope to advance.

To date, no systematic review has been found with or without a finished or ongoing meta-analysis addressing our investigative question, nor with similar inclusion criteria or with the same population scope. However, some literature reviews, such as that of Guerreiro and Monteiro [24], observed that occupational stress was one of the negative influencing factors on the quality of life of nursing workers. Freire and Costa [25] also analyzed that the work environment is conducive to health risks for nursing professionals and that such health risks interfere with the QWL.

Given the above, this study systematically analyzed the scientific evidence of the relationship between occupational hazards and quality of life and quality of work life among hospital workers.

## 2. Materials and Methods

A systematic review study was carried out according to the guidelines of the Preferred Reporting Items for Systematic Reviews and Meta-Analyses-PRISMA [26] guide and registered on the PROSPERO platform (CRD 2019127865). It assessed the relationship between occupational risks and the quality of work life of hospitals’ health workers. The investigative question was structured according to the PECO acronym: population (adult workers in hospitals), exposure (occupational risks), comparison (adequate control of occupational risks), and outcome (level of quality of work life).

The eligibility criteria were: (1) observational studies (cohort, cross-sectional, and case-control); (2) population studies of adult workers (from 19 to 65 years of age); (3) without date and language restriction; (4) studies with any type of instrument to assess QWL and QoL, as long as the instrument was validated; (5) any definition that the study presented about QWL and QoL; and (6) studies that presented the exposure and its relationship with the outcome.

### 2.1. Search in Database

The search was carried in June 2021 in the following databases: Latin American and Caribbean Center on Health Sciences Information (LILACS), MEDLINE/PubMed, PsycINFO, Cumulative Index to Nursing and Allied Health Literature (CINAHL), Scopus, Embase, and Brazilian Digital Library of Theses and Dissertations, Ovid, and a manual search in the bibliographic reference list of relevant studies and systematic reviews that addressed the topic of interest.

The descriptors and synonyms were defined in the Health Sciences Descriptors for the LILACS base, Subject headings Embase for Embase, and Medical Subject Heading for the other bases. The terms of the exhibition were: ergonomics, muscle problems, hazardous waste, occupational health, containment of biological risks, biological factors, occupational risks, occupational biological risks, risk factors, occupational accidents, risk management, safety management, occupational exposure, occupational noise, needle stick injury.

The terms for the population were: hospital directors, hospital anesthesia service, hospital janitorial services, central hospital warehouse, hospital dental team, hospital administrators, hospital cleaning service, hospital clinical staff, hospital nursery, hospital pharmacy service, hospital physiotherapy service, hospital nutrition service, occupational health nursing, health professional, hospital laundry service, hospital surgical center. The terms of the outcome were: quality of life, work-life balance, job satisfaction, workers’ compensation, well-being, and SF-36. All terms and their English synonyms were used with the Boolean operators AND and OR using all their combinations.

### 2.2. Selection of Studies, Extraction, and Data Analysis 

The studies were exported to the EndNote web, and duplicates were identified and removed. Two independent reviewers read the titles and abstracts. Studies that did not meet the eligibility criteria were excluded.

The selected studies were read in full to identify those eligible for the investigation. Disagreements between reviewers were discussed and solved by consensus, and when necessary, a third reviewer’s advice was sought. The information of the included studies was recorded in a spreadsheet prepared in Microsoft Excel, version 2010, with the following data: title, author, year of publication, country, study’s objective, type of study, sample, study duration, type of hospital, main sample characteristics, evaluated exposure variables, instruments used to assess exposure and outcome, primary results, and the main conclusions and limitations of the study.

The results of the studies were not combined through meta-analysis due to the considerable heterogeneity between the studies, both concerning the classification of exposure (total quality of life, mental health, physical health, among others) and the outcome (workload, stress at work, conflicts, overload, among others).

Thus, we performed a qualitative analysis describing the results and summarizing them according to the fulfillment of the results of the studies. To this end, we did not apply restrictions on the risk of bias or study design. The synthesis of the effects for each study in this review was based on the vote count of the association between exposure and outcome. The results with general characteristics, presented individually in a table, grouped the studies included in the review [27]. In this systematic review, both studies that assessed QoL focused on health workers, as long as they were associated with occupational risks, and studies that specifically assessed QWL were considered. Thus, the findings were independently summarized according to the type of questionnaire used.

### 2.3. Assessment of Methodological Quality and Risk of Bias

Two independent reviewers assessed methodological quality using the Newcastle–Ottawa scale (NOS), adapted for cross-sectional studies [28]. NOS is divided into three blocks using a star system (0—worse to 9—better) to score the studies: selection (a maximum of 5 stars), comparability (a maximum of 2 stars), and result (a maximum of 3 stars). The method recommended by Bernardo [29] was followed for the classification of methodological quality, which considers a total score above six stars as a high methodological quality, representing better quality.

The risk of bias was assessed by the Research Triangle Institute Item Bank (RTI-Item Bank) and two independent reviewers. Among the 29 RTI questions, those used in this review were: (i) clearly defined inclusion/exclusion criteria; (ii) use of valid and reliable measures to assess inclusion/exclusion criteria; (iii) the participants’ recruitment strategy was the same in the study groups; (iv) the level of detail in the description of the intervention or exposure; (v) the outcome evaluators were blinded to the intervention or exposure status of the participants; (vi) exposure was assessed using valid and reliable measures; (vii) results were evaluated using valid and reliable measures; (viii) some significant primary result was missing in the results; (ix) statistical methods used to evaluate the results were appropriate for the data; (x) reliable results considering the limitations of the study; and (xi) identification of the funding source.

Thus, a high risk of bias was considered when the study obtained ≥3 answers classified as unclear or negative, moderate risk when up to two responses were classified as unclear or negative, and low risk of bias when no answer was considered clear or negative [30,31].

## 3. Results

### 3.1. Selection and Characterization of the Studies

**Figure 1:**
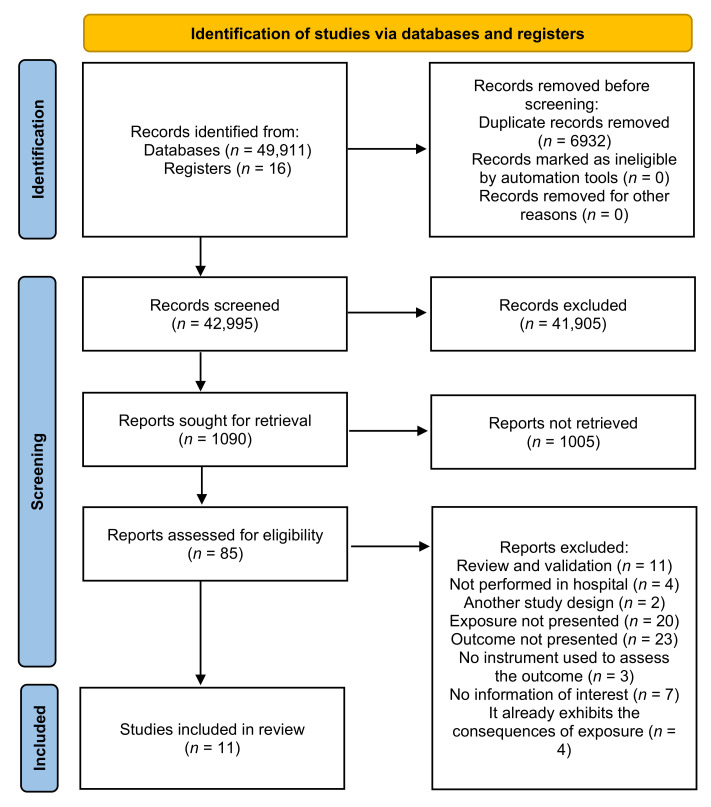
PRISMA flowchart of the study selection process.

The survey yielded 49,927 documents. Of these, 49,911 were based on the search in the databases, and 16 manually searched. Six studies were included in the review after excluding duplicates, reading titles and abstracts, and full reading [32,33,34,35,36,37]. One study was represented by two different publications [35,37]. Figure 1 shows the selection process.

The sample size of the studies by Lambert et al. [32], Makabe et al. [38], and Wu et al. [33] was significant; together, they totaled 8104 professionals distributed among nurses and doctors. The first two studies were multicentric, which contributed to the magnitude of the sample. The study with the lowest number of workers was Silva, Luz, and Gil [22], with only 35 workers distributed among the seven hospital sectors under study.

In total, this review included 6923 participants aged between 18 and 64 years of both genders. The general characteristics of the included studies are described in Table 1 and Table 2. Most of the studies (63.63%) were carried out in Asian countries [32,33,35,36,39,40,41], two were performed in Brazil [34,42], and other studies in Canada [35,37] and one in Australia [43]. The year of publication ranged from 2004 [32] to 2021 [39,40,41], and all studies were cross-sectional. Only the study by Nowrouziet al. [35,37] was qualitative and quantitative; the other studies were exclusively quantitative (Table 1 and Table 2).

The study by Lambert et al. [32] was the only one carried out in hospitals with different management modes (25 hospitals belonged to the private network, 3 were university hospitals, and 7 were government hospitals). Azevedo, Nery, and Cardoso [42] developed their study in a general hospital. The studies by Kalanlar, Akçay, and Karabay [41]; Silva, Luz, and Gil [34]; and Makabe et al. [38] were conducted in university hospitals, while in the studies by Almogbel [39], Foster, et al. [43], Ghasemi et al. [40], Kim and Kim [36], Nowrouzi et al. [35,37], and Wu et al. [33], the type of hospital was unclear.

All studies aimed, in general, to assess the factors associated with the quality of life at work of health professionals; however, in the study by Silva, Luz, and Gil [34] (noise) and Ghasemi et al. [40] (musculoskeletal complaints), the authors had already defined the exposure factor to be investigated. Makabe et al. [38] and Lambert et al. [32] also aimed to compare the findings between countries with different economic statuses and cultures, respectively (Table 1).

Health professionals were exclusively the target population in most of the studies included in this review. In six studies, nurses represented the sample. The studies by Ghasemi et al. [40] and Wu et al. [33] were carried out exclusively with doctors; Almogbel [39] investigated pharmacists; and Kalanlar, Akçay, and Karabay [41] included in their sample a multidisciplinary team (nurses, physicians, physiotherapists, psychologists, and social workers). The study of Silva, Luz, and Gil [34] was the only one performed with workers from different sectors, such as neonatal intensive care unit (ICU), nutrition, amphitheater, printing workshops, laundry, carpentry, and locksmiths. However, the study did not state which professionals from each sector made up the sample since these sectors allocated many professionals.

Female gender was the majority in 63.6% (*n* = 7) of the studies included in the review, and 18.2% (*n* = 2) of the studies did not present this information. Four studies did not show the average time the professionals worked in the hospital or average time in the profession. In the other seven studies, the average time in the profession or the time the professionals had worked in the hospital was >55 years (Table 1).

In this systematic review, only three occupational hazards were identified. Occupational noise was observed in the study by Silva, Luz, and Gil [34]; ergonomic risk (musculoskeletal discomfort) in the study by Ghasemi et al. [40]; and occupational stress in the other nine studies. 

### 3.2. Description of the Results of Studies That Evaluated Occupational Risks with the Quality of Life (QL) of Workers

Among the studies that evaluated the workers’ QoL, different instruments were used. Three studies used the abbreviated version of the World Health Organization Quality of Life scale (WHOQOL-Bref), two studies used the Medical Outcomes Study Questionnaire Short Form 36 Health Survey (SF-36), and one study used the Short Form SF-12v2, and the other the QWLS. Due to cultural and linguistic differences between countries, the authors translated, adapted, and validated the instruments before the questionnaires’ use. For example, the study by Almogbel [39] excluded a question about sexual activity because it was inappropriate for Saudi Arabia’s culture and could compromise the research.

The QoL categorization in the studies demonstrates the versatility of the scales used and the lack of a gold standard method for evaluating the construct. Foster et al. [43], Lambert et al. [32], and Wu et al. [33] considered QoL in two components: physical and mental. In the study by Makabe et al. [38], QoL was assigned from the average value of 80 points on the scale used, considering a score below this value as low QoL. Almogbel [39] considered high scores on the scale as better QoL. In the study by Silva, Luz, and Gil [34], QoL was organized into five classes (1: very dissatisfied; 2: dissatisfied; 3: neither satisfied nor dissatisfied; 4: satisfied; 5: very satisfied).

For occupational stress assessment, the scales used also varied. One study assessed exposure using the Nursing Stress Scale (NSS). The other four studies used other types of instruments. Silva, Luz, and Gil [34], to assess occupational risk in the hospital (noise), used equipment to measure noise levels and applied an adapted hearing habits questionnaire. 

The general questionnaires used to assess the quality of life have questions related to the scope of work among their questions. Given the heterogeneity observed concerning the instruments used to assess occupational risks and QoL, the relationship of occupational risks with the QoL of workers, especially with variables related to the work environment, was presented differently. Almogbel [39] found a mean total QoL of 55.5 (SD = 8.5), with the highest score in the social relationships domain (µ = 15.4; SD = 3.3) and average occupational stress of 43.85 (SD = 5.43) in all domains. When analyzing the relationship between QoL and occupational stress through multiple regression analysis, they found a negative and significant association (β = −0.454; 95% CI, −0.6096 to −0.212). In the study by Foster et al. [43], the authors found that the QoL of nurses working with mental health was 52.62 (SD = 8.30) for physical health and 43.59 (SD = 11.34) for mental health. Between the numbers of work-related stressors and QoL, there were significant negative correlations both for physical health (r = 0.111, *p* ≤ 0.05) and for mental health (r = 0.108, *p* ≤ 0.05). Additionally, those nurses who were younger and had fewer work-related stressors had higher mental health than nurses of the same age group and with more work-related stressors (*p* = 0.02). Age (*p* = 0.005) and years of work in the mental health area (*p* = 0.006) were also associated with mental health for those who reported ≥ 20 work-related stressors.

Lambert et al. [32] performed multiple regression, using a set of variables for each country, to identify which factors are associated with nurses’ QWL (physical and mental health). In Japan, the factors associated with physical health were workload 1.7% (*p* = 0.02) and the number of people in the household 3.3% (*p* = 0.01). In South Korea, these factors were the method used to cope with stressors at work by searching for social support, representing 8.1% (*p* = 0.01), and the probability of leaving the profession, representing 15% (*p* = 0.013).

In Thailand, the variables associated with physical health were the number of people at home (3.2%) (*p* = 0.002), the number of years working as a nurse (5.5%) (*p* = 0.008), and family income (6.8%) (*p* = 0.028). In the study carried out in Hawaii, the associated factors were workload (6.2%) (*p* = 0.000), the probability of leaving the profession (7.8%) (*p* = 0.006), higher educational level (9.1%) (*p* = 0.013), and forms of coping in the profession (10.2%) (*p* = 0.017).

In the regression models used to identify factors associated with the mental health of Japanese nurses, the probability of leaving the profession (8.6%) (*p* = 0.000), lack of support (12.8%) (*p* = 0.000), and ways of coping in the profession (16%) (*p* = 0.000) were the identified variables. In South Korea, the associated factors were age (21%) (*p* = 0.000), distancing (27.2%) (*p* = 0.012), workload (32.5%) (*p* = 0.015), probability of leaving the position of nurse (36.6%) (*p* = 0.030), and planning for problem solving (39.9%) (*p* = 0.042).

Among Thai nurses, conflict with doctors (8%) (*p* = 0.000), probability of leaving the profession (13.5%) (*p* = 0.000), ways of coping in the profession (18.6%) (*p* = 0.000), seeking social support (20.1%) (*p* = 0.015), and lack of support (21%) (*p* = 0.046) were the main identified associated factors. On the other hand, in Hawaii, the variables were forms of coping in the profession (17.3%) (*p* = 0.000), conflict with other nurses (24.5%) (*p* = 0.000), probability of leaving the profession (28.4%) (*p* = 0.000), positive reassessment (30.6%) (*p* = 0.000), lack of support (32%) (*p* = 0.002), distancing (32.8%) (*p* = 0.011), and workload (33.6%) (*p* = 0.014).

The study by Makabe et al. [38] also presented some variables significantly related to the quality of life, such as the ability to deal with stress (β 0.44; *p* < 0.01), social support (β 0.21; *p* < 0.01), and stress at work (β −0.07; *p* < 0.01) (adjusted R^2^ = 0.46). Likewise, Silva, Luz, and Gil [34] concluded that QoL considered regularly by professionals was a reflection of the unhealthiness presented in the sectors, since both the minimum and maximum noise levels in all the sectors evaluated, except for the neonatal ICU, exceeded what is provided for in the legislation for the hospital environment. The authors found that the noise levels and the physiological consequences were responsible for the workers attributing regular values to the domain quality of life. In conclusion, they noted the need to implement a Hearing Conservation Program. There was no difference between the studied sections regarding the workers’ QoL, classified between regular and good by the majority.

Wu et al. [33] identified that physical effort, psychological stress, insufficient function, working time, overload in the function, occupation, recognition, and working in the surgery department were the main factors. They were associated with influencing the doctor’s quality of life (both physical and mental components). The physical environment, function overload, and occupation were related only to the physical component. The authors concluded that doctors’ quality of life was influenced by occupational and behavioral factors, coping resources, and age.

Considering the assessment of methodological quality, all studies presented high quality ranging from 7 to 9 points (>6 points as recommended by Bernardo [29]). The issues that most contributed to high methodological quality were the selection process used in the studies, satisfactory response rate, use of validated tools, clearly described, and appropriate statistical tests performed, mostly with the level of probability (*p*-value) (Appendix A Table A1).

Three studies included in this review [34,39,43] were classified as high risk of bias, and the lack of clarity in the information contributed most to this result. For example, Silva, Luz, and Gil [35] did not present the inclusion/exclusion criteria of the participants or the sample calculation process. In addition, the participants volunteered to participate, which may have influenced the answers about the quality of life. The other three studies [32,33,38] had a moderate risk of bias (Appendix A Table A1).

### 3.3. Description of the Results of Studies That Evaluated Occupational Risks with the Quality of Life at Work (QWL)

Different instruments were used to assess QWL. Kim and Kim [36] and Nowrouzi et al. [35,37] used the Work-Related Quality of Life Scale (WRQoL), and the others [40,41,42] used other scales (QWLS, TQWL-42, and Walton’s 35-item tool). 

The categorization for QWL was also presented differently between the studies. Kim and Kim [36] categorized QWL into compassion/satisfaction, secondary traumatic stress, and exhaustion. Nowrouzi et al. [35,37] categorized QWL as high (scores 4 and 5) or low (scores 1 to 3); Azevedo, Nery, and Cardoso [42] categorized it as unsatisfactory (scores 0 to 50) or satisfactory (scores 50.01 to 100); Ghasemi et al. [40] classified it as low (scores < 58), moderate (scores between 59–118), or high (scores > 118); and Kalanlar, Akçay, and Karabay [41] considered high scores on the scale as representing better QWL.

Regarding the instruments used to assess occupational stress exposure considered for this review, each of the four studies used different scales. Ghasemi et al. [40], who assessed another occupational risk, used the Nordic Musculoskeletal Questionnaire (NMQ) to assess complaints of pain and musculoskeletal discomfort, in addition to evaluating posture through the application of the Rapid Entire Body Assessment (REBA).The relationship between occupational risks and QWL was presented differently by the studies. Analyzing the association between occupational stress and QWL, Azevedo, Nery, and Cardoso [42] found, in the univariate analysis, that workers with the worst perception of QWL were those who, according to the quadrants of the demand-control model, perceived their work as active work (PR = 2.40; 95% CI: 1.44–4.02; *p* < 0.001) and high strain (RP = 3.36; 95% CI: 2.04–5.56; *p* < 0.001). In the model adjustment, both active work (RP = 1.74; 95% CI: 1.04–2.92; *p* = 0.034) and high strain (RP = 0.54; 95% CI: 1.51–4.27; *p* < 0.001) were associated with the perception of dissatisfaction with QWL.

The only study identified that assessed ergonomic risk [40] found that QWL was a significant predictor of pain and discomfort in the neck (OR: 0.99; 95% CI: 0.98–0.99), shoulder (OR: 0.99; 95% CI: 0.99–1.00), upper back (OR: 0.99; 95% CI: 0.98–0.99), elbows (OR: 0.99; 95% CI: 0. 99–1.00), and legs (OR: 0.99; 95% CI: 0.99–1.00).

In Turkey, Kalanlar, Akçay, and Karabay [41] found a positive correlation between perceived stress at work and QWL (*p* ≤ 0.05). The highest QWL score was obtained in the working conditions dimension (3.47), and the lowest score for the stress dimension (1.34), and the average score of the perception of stress at work was 33.18 (SD = 63.29).

Kim and Kim [36] found that the relationship between work stress and secondary traumatic stress (r = 0.28; *p* = 0.001) and work stress with burnout (r = 0.33; *p* = 0.001) showed a statistically significant positive correlation; that is, the greater the stress at work, the greater the secondary traumatic stress and burnout.

Nowrouzi et al. [35,37] identified that nurses with a total stress score greater than 65 were five times more likely to have low QWL (*p* < 0.01). Still, the WRQoL and NSS subscale analyses were not statistically significant with the variables of interest used concerning high or low QWL.

Concerning methodological quality assessment, all studies also presented high quality, ranging from 7 to 9 points, as established by Bernardo [29]. As observed in studies that evaluated QoL, the issue that most contributed to high methodological quality in studies that evaluated QWL was the selection process used. Therefore, the response rate was satisfactory, in addition to the statistical tests used and the use of validated tools.

Four studies included in this review [36,40,41,42] were classified as having a high risk of bias, where a lack of clarity in the information most contributed to this result. Only one study [35,37] presented a moderate risk of bias (Appendix A Table A1). The reason for the study by Kim and Kim [36] having a high risk of bias was the sample selection process, as the authors did not present in detail the criteria for inclusion/exclusion of participants, in addition to a lack of information, such as a declaration of financing. On the other hand, Kalanlar, Akçay, and Karabay [41] did not make it clear how the inclusion/exclusion process of the study participants took place, the details in the description of both occupational stress and QWL were unclear, and the statistical analysis used we consider partially appropriate for the data, in this sense, we judged the same with a high risk of bias; thus, the results are partially reliable considering the limitations presented by the study. The study by Nowrouzi et al. [35,37] presented the result of a survey carried out with professionals from rural regions, which may be different compared to the metropolitan area.

## 4. Discussion

From the research question used to carry out this review, systematic methods and protocols from international institutions were followed so that quality studies were identified and analyzed. In this way, it was possible to identify the articles contributing to demonstrating the relationship between occupational risks with quality of life and quality of work life in a hospital environment. 

The synthesis of the evidence demonstrated that most of the studies identified health professionals as the target population. Only one study [34] involved other professionals; however, their characteristics were not considered in the analysis. Different professionals are required, and the occupational risks may be different with the development of the function. Therefore, the functions of the professionals allocated in each area have not been considered in the study, and it is a significant limitation.

Understanding the relationship of occupational risks with both QoL and QWL of hospital workers is extremely important for the actions of managers and public authorities to be exercised. All studies included in this review presented variables related to the work environment associated with occupational risk and the perception of QoL and QWL. Considering only the nine studies that investigated occupational stress, it is evident that the investigation methods adopted, both for occupational risk and for QoL and QWL, were heterogeneous and without standardization, regardless of which instrument was used. Thus, the fact that each study considered different variables combined with the use of divergent instruments contributed to making it difficult to establish associations between what is really related to QoL and QWL of workers.

Considering that the assessment of QoL and QWL is not simple to perform, the way to categorize or analyze the domains that influence the two constructs is also diversified. In the studies included in this review, the instruments used to assess both QoL and QWL were generic, according to the division established by Ferraz [44]. The fact that each study uses a type of instrument to assess the constructs, even though all of them have been validated, proves the inexistence of a gold standard method for assessing them, regardless of the labor sector. Many authors consider this scope due to two main reasons: first, QWL cannot be isolated from life outside the organization; therefore, other dimensions of life must be considered in the analysis process. Secondly, there are multiple concepts and different theoretical models about the constructs [45,46,47].

Additionally, the divergence in the objectives that guided the studies; the way they categorized occupational stress, quality of life, and QWL; the adoption of different concepts about the constructs; the measures of analysis used; and the conclusions are a series of limitations that make it difficult to make associations between studies, in addition to the need to interpret these results with caution. It reinforces the lack of observational studies with better development involving both QoL and QWL.

A study on QoL, whether QWL or in other dimensions of life, demands prudence in its performance and, above all, in its interpretation. Quality of life at work can be influenced by numerous factors that go beyond organizational issues, such as the political landscape of the country, socioeconomic status, family relationships, culture, and religious and traditional customs. These points can influence their association with organizational factors and with life as a whole. Thus, the studies included in this review that assessed occupational stress considered some of these variables in the analysis process: family issues, income, education, marital status, age, cultural aspects, etc.

Furthermore, the multicenter studies included in this review aimed to assess whether occupational stressors were related to QoL among countries with cultural and economic differences. It was possible to identify that the factors that cause occupational stress and contribute to the worker’s perception of their QOL do not differ according to location. Makabe et al. [38] found that countries that have high technologies, and therefore are richer, were not enough for workers to improve their QoL. Similarly, Lambert et al. [32] identified that the country’s characteristic of constant conflict between doctors and nurses was the one that most contributed to the lowest QoL scores.

Only three studies [32,36,43] considered the time nurses had worked in the hospital to analyze whether such time affected QoL and QWL. Assuming that the consequences of many occupational risks, especially occupational stress, occur due to the time of exposure, then assessing the influence of this variable on QWL is extremely important for establishing long-term measures.

Occupational stress was related to QoL and QWL in most of the studies included in this review. The studies showed that dealing with occupational stress is crucial to maintaining adequate QoL and QWL. They also pointed out that high workload and working hours are the leading occupational stressors influencing QoL and QWL. The factors associated with occupational stress were significant risk factors for the workers’ quality of life [32,33,35,36,38,39,41,42,43].

In the study by Silva, Luz, and Gil [34], the authors verified that occupational noise is an occupational risk that presents an essential contribution to low or regular perception of quality of life among professionals. The authors found that the nutrition sector was the only one that presented, in most cases, the lowest score for the QWL domains, that is, poor quality of life, in addition to being the second sector with the highest noise intensity. Although only one study has evaluated this occupational risk, the fact that it was carried out in different hospital sectors shows how present this risk is in working life. It also indicates the need for further studies, separately between sectors and categorizing the different professions that comprise it.

The QWL of surgeons in the study by Ghasemi et al. [40] was moderate, with the best domain referring to the opportunities created by work to learn, acquire, and apply new skills and knowledge. The worst domain was the one referring to the balance between professional life, leisure, and family. QWL was associated with musculoskeletal pain in various parts of the body, which reinforces the performance of repetitive work, increased time in certain positions, and increased demands of manual skill among these professionals. However, the study had significant limitations, such as a small sample and it did not investigate important variables for QWL, such as regular exercise, job satisfaction, and job stress, so the results need to be interpreted with caution.

Even though an exhaustive investigation was carried out in this review to identify all eligible studies that addressed the investigative question and also adopting a strict methodology in its development, it still has limitations. Some of the limitations are the inclusion of studies with hospital workers only and not from other health places, such as private clinics and offices; and the non-inclusion of studies in which the sample already suffered the consequences of occupational risks, such as repetitive strain injury (RSI), musculoskeletal disorders, and illnesses resulting from occupational accidents, among others. Finally, the study design presented in all the papers makes it impossible to observe a cause–effect relationship between occupational risks and the QWL.

In general, the existing synergy between the QoL and QWL constructs makes their application a fine line in the differentiation process, which is why we observed studies that used these concepts as synonyms. Perhaps this misunderstanding occurs to the detriment of the difficulty in assessing the quality of life at work without considering the quality of life beyond the physical barriers of organizations. Thus, the findings of this systematic review reinforce the complexity in the authors’ use of the construct. Thus, researchers’ efforts are needed to conduct more complex studies that, above all, take into account the quality of life at work construct when the intention is to investigate the dimensions of work in the health outcomes (physical and/or mental) of workers.

Regarding the instruments used to assess occupational stress, the used scales also varied. Two studies assessed exposure using the Nursing Stress Scale (NSS); seven studies used other independent scales. Silva, Luz, and Gil [34] used equipment to measure noise levels to assess occupational risk in the hospital and applied an adapted hearing habits questionnaire. Ghasemi et al. [40] used the Nordic Musculoskeletal Questionnaire (NMQ) to assess complaints of musculoskeletal pain and discomfort, in addition to evaluating posture through the application of the Rapid Entire Body Assessment (REBA).

The way quality of work life was categorized in the studies demonstrates the versatility of the used scales and the lack of a gold standard method for evaluating the construct. In the study by Kim and Kim [36], QWL was categorized into compassion/satisfaction, secondary traumatic stress, and exhaustion. Foster et al. [43], Lambert et al. [32], and Wu et al. [33] considered QWL in two components: physical and mental. In the study by Makabe et al. [38], QWL was assigned from an average value of 80 points on the used scale, considering low QWL as a score below this value. Nowrouzi et al. [35,37] categorized QWL as high (scores 4 and 5) or low (scores 1 to 3); Azevedo, Nery, and Cardoso [42] as unsatisfactory (scores 0 to 50) or satisfactory (scores 50.01 to 100); Ghasemi et al. [40] s low (scores < 58), moderate (scores between 59–118), or high (scores > 118). Almogbel [39] and Kalanlar, and Akçay, and Karabay [41] considered high scores on the scale as representing better QWL. In Silva, Luz, and Gil [34], QWL was organized into five classes (1: very dissatisfied; 2: dissatisfied; 3: neither satisfied nor dissatisfied; 4: satisfied; 5: very satisfied).

## 5. Conclusions

The studies included in this systematic review presented a series of occupational and demographic variables that demonstrated a relationship between occupational risks with quality of life and QWL. Therefore, this suggests that labor factors are essential influencers for these professionals, leading them to consider their quality of work life as low or regular. However, the quantity, methodological quality, and design of primary studies on the relationship proposed here is still limited and do not allow a summary of them.

Although the results of this review should be interpreted with caution due to the quality of the studies included, the high methodological quality applied in conducting the review reinforces the validity of the results. In this sense, the findings of this review positively contribute to practice, mainly due to the growing number of institutions that show interest in improving the quality of life of their workers and favors the expansion of knowledge on the addressed subject. This review presents information about the work environment that managers can modify to prevent and/or minimize the consequences of occupational stress. Some actions that could be performed are enriching and enhancing functions; offering adequate working conditions, training, skill development, adequate working hours, incentives for professional advancement, compensation plans, and psychological and social support; and adopting quality of life programs at work, among other measures.

In light of the current state of evidence, further studies are required, especially studies that are more homogeneous in terms of the concept of quality of life and quality of work life, the use of instruments, the forms of analysis, and the categorization of the construct. It is also necessary for investigators to emphasize studies focused on other professionals working in the hospital and not just on/in health professionals. In addition, caution should be exercised as researchers should always add variables to the organizational and/or health issues in developing studies, such as cultural, religious, political, occupation time in the profession, and/or current work.

## Figures and Tables

**Table 1:** Summary of the findings in the documents included in the review regarding quality of life (QoL).

Author/Year	Country	Objective	Study’s Design	Instrument to Assess QoL	Instrument to Assess the Occupational Risk	Occupational Risk Assessed	Sample Size (Occupation)	Gender	Occupation Time (Years)
Almogbel, 2021 [39]	Saudi Arabia.	Evaluate the association between pharmacists’ Quality of life (QOL) and occupational stress in Saudi Arabia.	Cross-sectional	World Health Organization Quality of Life–Brief scale (WHOQOL-BREF).	Effort-Reward Imbalance (ERI).	Occupational stress.	204 (pharmacists).	Male: 61.2%; Female: 38.8%.	Mean 8.1 (SD = 7.2).
Foster et al., 2020 [43]	Australia.	Identify the health-related Quality of life of mental health nurses (HR-QoL) and work-related stressors; associations between stressors and HR-QoL; and HR-QoL predictors.	Cross-sectional	Short Form SF-12v2-12-item.	Work-related stressor items were informed by literature and a prior pilot study. Work-related stressors were grouped in three categories comprising 23 different stressors: (1) Consumer/Carer stressors, (2) Collegial included staff behaviors and relationships in the multidisciplinary team and (3) Organizational included the nursing role and organizational resources.	Occupational stress.	498 (nurses).	Male: (26%); Female: (74%);	<1–4 years (18%); 5–9 years (17%); 10–14 years (15%); >14 (50%).
Lambert et al., 2004 [32]	Japan, South Korea, Thailand, and the USA (Hawaii).	Culturally compare factors contributing to nursing shortages in countries that produced a limited number of research findings on stress in nurses.	Cross-sectional	SF-36 Health Survey (SF-36).	Nursing Stress Scale (NSS).	Occupational stress.	1.554 (nurses).	Female: 93.2% (Japan); 98.7% (South Korea); 94.6% (Thailand); 93.4% (USA).	Average:11.8 (Japan); 8.01 (South Korea); 11.7 (Thailand); 13.4 (USA).
Makabe et al., 2018 [38]	Japan, Singapore, Malaysia, Thailand, and Bhutan.	Compare nurses’ Quality of life and investigate the main determinants among Asian countries with different economic statuses.	Cross-sectional	World Health Organization Quality of Life (WHOQOL-Bref).	NIOSH Questionnaire.	Occupational stress.	1201 (nurses in Japan); 1040 (nurses in Singapore); 1001 (nurses in Malaysia); 418 (nurses in Thailand); 169 (nurses in Bhutan).	Female: 93% Japan; 93% Singapore; 94% Malaysia; 97% Thailand; 70% Bhutan.	Average: 15 (Japan); 08 (Singapore); 05 (Malaysia); 17 (Thailand); 08 (Bhutan).
Silva, Luz and Gil, 2013 [34]	Brazil.	Assess noise levels in different hospital environments and investigate the impact of this exposure on the Quality of life of professionals working in these environments.	Cross-sectional	World Health Organization Quality of Life (WHOQOL-Bref).	On-the-spot measurement of sound pressure levels, the minimum value is the weakest intensity, and the maximum as the strongest sound pressure intensity in each sector.	Occupational noise.	Seven sectors of the hospital and 35 workers (five from each sector).	Features only of sectors.	Features only of sectors
Wu et al., 2010 [33]	China.	Assess doctors’ quality of life and explore their main influencing factors, especially demographic characteristics, behavioral, occupational factors, and coping resources.	Cross-sectional	SF-36 Health Survey (SF-36) Chinese version.	Occupation Stress Inventory-Revised Edition (OSI-R) Chinese version.	Occupational stress.	2721 (Physicians).	Male: 37.6%; Female: 62.4%.	No information.

**Table 2:** Summary of the findings in the documents included in the review regarding quality of work life (QWL).

Author/Year	Country	Objective	Study’s Design	Instrument to Assess QWL	Instrument to Assess the Occupational Risk	Occupational Risk Assessed	Sample Size (Occupation)	Gender	Occupation Time (Years)
Azevedo, Nery and Cardoso, 2017 [42]	Brazil.	Analyze the association between occupational stress, Quality of work life and associated factors among nursing workers	Cross-sectional	Total Quality of Work Life–TQWL-42.	Job Stress Scale (JSS).	Occupational stress.	309 (nurses = 38.5%; nursing technician = 53.4%; nursing assistant = 8.1%).	Male: 11%; Female: 89%.	Mean 7.1.
Ghasemi et al., 2021 [40]	Iran.	Evaluate QWL among surgeons and investigate its association with musculoskeletal complaints.	Cross-sectional	Walton’s 35-item questionnaire.	Nordic Musculoskeletal Questionnaire (NMQ) and Rapid Entire Body Assessment (REBA).	Musculoskeletal complaints.	74 (surgeons).	Male: 60.8%; Female: 39.2%.	Mean 7.00 (SD = 4.23).
Kalanlar, Akçay and Karabay, 2021 [41]	Turkey.	Examine the relationship between the Quality of working lives and the perceived stress of health personnel working in a hospital specialized.	Cross-sectional	Quality of Work Life Scale (QWLS).	Perceived Stress Scale (PSS).	Occupational stress (perceived Stress).	80 (nurses, physicians, physiotherapists, psychologists and social workers).	Male: 31.3%; Female: 68.7%.	≤10 years (23.7%); 11–20 years (43.8%); ≥21 years (32.5%).
Kim and Kim, 2017 [36]	South Korea.	Identify the emotional work, work stress, and QWL of hospital nurses; examine the correlation between them and analyze the factors that affect the Quality of professional life.	Cross-sectional.	Korean version of the Professional Quality of Life Scale (satisfaction of compassion/subscale Fatigue version 5).	Clinical tool developed by Ku and Kim (1984).	Occupational stress.	136 (nurses).	No information.	Mean 10.71 (SD = 8.11). <5 years (30.1%); 5~10 years (21.3%); 11~20 years (30.9%); >20 years (17.6%).
Nowrouzi et al., 2015 [35,37]	Canada.	Examine the QWL of nurses working in midwifery wards at four hospitals in northeastern Ontario and explore factors that influence their QWL.	Cross-sectional	Work-Related Quality of Life Scale (WRQoL).	Nursing Stress Scale (NSS).	Occupational stress.	111 (nurses).	Male: 5.4%; Female 94.6%.	Mean 11.6 (SD = 9.01). <35 years (24.4%); 35–44 years (35.3%); 45–54 years (23.2%); ≥55 years (17.1%).

## Data Availability

The study did not report any data.

## Appendix A

**Table A1:** Assessment of Methodological Quality and Risk of Study Bias.

Study	Methodological Quality				Bias’ Risk			
	Selection	Comparability	Result	Total	Yes	No	Unclear	Result
Almogbel, 2021 [30]	* * * * *	* *	* *	9	7	1	3	High risk
Azevedo, Nery and Cardoso, 2017 [33]	* * * * *	* *	* *	9	5	2	4	High risk
Foster et al., 2020 [34]	* * * * *	*	* *	8	6	2	3	High risk
Ghasemi et al., 2021 [31]	* * * * *	*	* *	8	7	1	3	High risk
Kalanlar, Akçay and Karabay, 2021 [32]	* * * * *	-	* *	7	3	1	7	High risk
Kim and Kim, 2017 [27]	* * * * *	*	* *	8	4	3	4	High risk
Lambert et al., 2004 [23]	* * * * *	*	* *	8	6	3	2	Moderate risk
Makabe et al., 2018 [29]	* * * * *	*	*	7	7	2	2	Moderate risk
Nowrouzi et al., 2015 [26,28]	* * * * *	*	* * *	9	7	2	2	Moderate risk
Silva, Luz and Gil, 2013 [35]	* * * * *	*	* *	8	4	4	3	High risk
Wu et al., 2010 [24]	* * * * *	*	* *	8	6	2	3	Moderate risk

* refer to the number of points obtained in each of the components (Selection, Comparability, Result) of the Newcastle-Ottawa Scale.
